# A Hybrid Approach Based on Seasonal Autoregressive Integrated Moving Average and Neural Network Autoregressive Models to Predict Scorpion Sting Incidence in El Oued Province, Algeria, From 2005 to 2020

**DOI:** 10.34172/jrhs.2023.121

**Published:** 2023-09-29

**Authors:** Safia Zenia, Mohamed L’Hadj, Schehrazad Selmane

**Affiliations:** ^1^L’IFORCE, SPA, Higher National Veterinary School of Algiers, Algeria; ^2^Scorp2, BeniMessous University Hospital Centre, Ministry of Health, Algeria; ^3^L’IFORCE, Scorp2, Faculty of Mathematics, University of Sciences and Technology Houari Boumediene, Algeria

**Keywords:** El Oued province, Neural network autoregressive model, Prediction, SARIMA model, Scorpion sting

## Abstract

**Background:** This study was designed to find the best statistical approach to scorpion sting predictions.

**Study Design:** A retrospective study.

**Methods:** Multiple regression, seasonal autoregressive integrated moving average (SARIMA), neural network autoregressive (NNAR), and hybrid SARIMA-NNAR models were developed to predict monthly scorpion sting cases in El Oued province. The root mean square error (RMSE), mean absolute error (MAE), and mean absolute percentage error (MAPE) were used to quantitatively compare different models.

**Results:** In general, 96909 scorpion stings were recorded in El Oued province from 2005-2020. The incidence rate experienced a gradual decrease until 2012 and since then slight fluctuations have been noted. Scorpion stings occurred throughout the year with peaks in September followed by July and August and troughs in December and January. Sting cases were not evenly distributed across demographic groups; the most affected age group was 15-49 years, and males were more likely to be stung. Of the reported deaths, more than half were in children 15 and younger. Scorpion’s activity was conditioned by climate factors, and temperature had the highest effect. The SARIMA(2,0,2)(1,1,1)_12_, NNAR(1,1,2)_12_, and SARIMA(2,0,2)(1,1,1)_12_-NNAR(1,1,2)_12_ were selected as the best-fitting models. The RMSE, MAE, and MAPE of the SARIMA and SARIMA-NNAR models were lower than those of the NNAR model in fitting and forecasting; however, the NNAR model could produce better predictive accuracy.

**Conclusion:** The NNAR model is preferred for short-term monthly scorpion sting predictions. An in-depth understanding of the epidemiologic triad of scorpionism and the development of predictive models ought to establish enlightened, informed, better-targeted, and more effective policies.

## Background

Scorpions are among the oldest known arthropods. They appeared in aquatic environments 450 million years ago. They are found on all continents except Antarctica and predominately live in deserts and have adapted to a broad variety of climatic and environmental conditions. Their activity is related to climatic conditions; they are active in spring and summer and enter into hibernation at the beginning of autumn; however, some species are active during the cold season.^1,2^ In the updated list of all extant scorpion species, 2769 species are catalogued in the world.^3^ The Buthidae and Hemiscorpiidae families include the 30 documented species potentially lethal to humans.^2^

Scorpion sting and the resulting envenomation are referred to as scorpionism. Scorpionism represents a public health problem in affected tropical and subtropical regions, including North-Saharan Africa, Sahelian Africa, South Africa, and the Near and Middle East. Some scorpion species induce severe clinical symptoms, fatal at times. Scorpionism is a multi-factor phenomenon; geographical location, socio-economic structure of the society, scorpion species, and climate are among the driving factors.^4^

Scorpionism, a common and dreaded event, was recognized in the mid-1980s as a public health problem in Algeria due to the morbidity and mortality it sources and the financial burden it enforces. The population at risk of scorpion sting is constantly growing; it has increased from 30% in 1997 to 86% in 2019.^5^

 To enhance the current monitoring strategies and establish enlightened, informed, better-targeted, and more effective policies, an in-depth understanding of the epidemiologic triad of scorpionism, along with the supply of reasonable and reliable predictions, is necessary.

 The present study was designed to assess the epidemiological and demographic characteristics of stung people, find out climate factors associated with scorpion activity, and develop models. In addition, the study aimed to compare the performance of the mentioned models using a hybrid approach based on seasonal autoregressive integrated moving average (SARIMA) and neural network autoregressive (NNAR) models to predict monthly scorpion sting cases in El Oued; a province of the South-East Algeria, which records a high number of scorpion stings. To the best of our knowledge, this is the first time to apply this hybrid approach to scorpion sting data.

## Methods

###  Study region

El Oued, a Saharan province of Algeria, is located in the Oriental Erg region between 32°00’ N and 34°30’ N and 5°11’ E and 9°04’E. Made up of 30 municipalities. It covers an area of 44 586 km^2^ and an estimated population of 925 000 inhabitants as of 2020. The relief of the province is characterized by a sandy region that presents itself under a double aspect; the Erg and the Sahara, a form of rocky plateaus that extends towards the South with an alternation of dunes and rocky ridges and a region of depression characterized by the presence of a multitude of chotts that plunge towards the East, representing a conducive environment for scorpion species. The province has a subtropical desert climate with extremely hot and dry summers and mild winters with light and sporadic precipitations.

###  Data

 Monthly scorpion sting data from January 2005 to December 2020, provided by the Directorate of Health and Population (DHP) of El Oued province, included gender, age, month, year, and residence of the victims.

Monthly average (T), maximum (Tmax), and minimum (Tmin) temperature (°C), monthly average relative humidity (RH) (%), monthly precipitation amount (Pr) (mm), monthly average wind speed (W) (km/h), and atmospheric pressure at sea level (SLP) (hPa) were extracted from climate data (Available from: https://en.tutiempo.net/climate/ws-605590.html accessed on March 1, 2022).

 This study was approved by DPH; the proposal was sent on January 2, 2022, and was approved on February 2, 2022.

###  Statistical analysis and modeling

Descriptive statistics were used to analyze the quantitative data. A univariate regression analysis was performed to determine any significant relationship between climate factors and scorpion stings. The multiple regression, SARIMA, SARIMAX, NNAR, and hybrid SARIMA-NNAR models were developed to find out the best-fitting models.^6,7^ A description of all utilized statistical approaches is given in Supplementary file 1. Statistical analyses and model equation estimations were performed using R software (version 3.4.4; Network Theory Ltd., Bristol, UK), SPSS (version 26.0), and EViews 12. Maps were produced using GeoDa software (https://geodacenter.github.io/). The significance level was set to 5%.

The root mean square error (RMSE), mean absolute error (MAE), and mean absolute percentage error (MAPE) were estimated as prediction performance to measure the closeness of the observed values to the predicted values, and the Pearson product-moment correlation coefficient (r) was estimated to measure a linear correlation between two variables (definitions are provided in Supplementary file 1).

## Results

###  Exploratory data analysis

**Figure 1:**
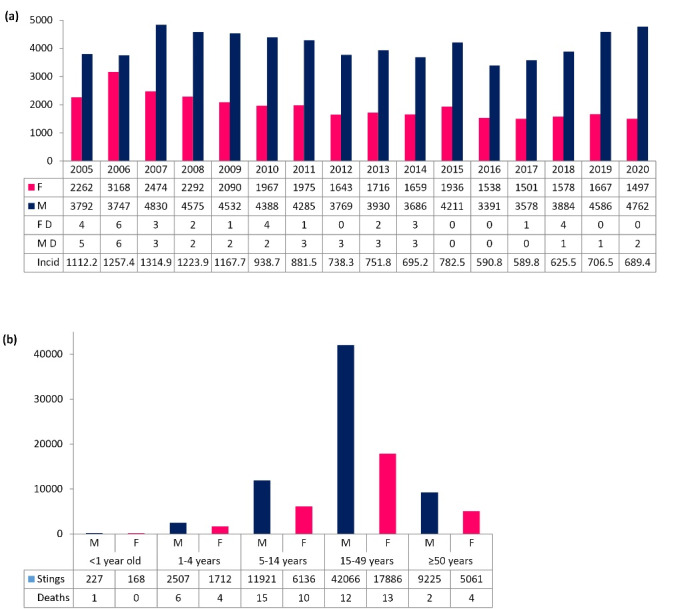
Demographic features of scorpion stings in El Oued province: (a) Yearly evolution of scorpion stings, deaths (FD: Female deaths; MD: Male deaths) and incidence rate per 100 000 inhabitants (Incid) and (b) sting cases stratified by the age group. Note. Blue bars: The number of stung males; Pink bars: The number of stung females

The number of scorpion sting cases recorded in El Oued province between January 1, 2005, and December 31, 2020 reached a total of 96909 with a mean ± standard deviation (SD) of 6057 ± 691 cases per year (95% confidence interval [CI]: 5809.70, 6304.30). The distribution by year and gender of scorpion sting cases and deaths, as well as the incidence rate, are displayed in Figure 1a. In 16 years, the lethality, defined as the ratio of the number of deaths attributed to scorpion envenomation to the number of scorpion stings over the same period expressed as the percentage, has been reduced by 81.6% from 0.17% in 2006 to 0.03% in 2020. There was a significant decrease in the incidence rate with a peak observed in 2007 of 1315 cases per 100 000 inhabitants compared to 689 cases per 100 000 inhabitants recorded in 2020. The scorpion stings were more frequent in the male population (68%), and the most affected age group for both genders was 15-49 years with 61.86% of cases (Figure 1b). Of the 67 recorded deaths, 36 were males, and children under the age of 15 counted 36 (54%) deaths with a male-to-female ratio of 1.4 of whom, 11 (16%) cases were under the age of 5. Of the 22 671 (23.4% of all sting cases) stung children under 15 years of age, 65% were boys and 35% were girls.

**Figure 2:**
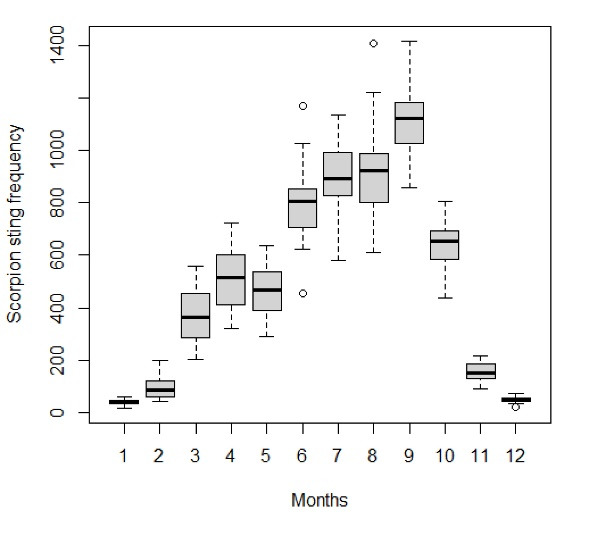
Box plot with monthly average of recorded scorpion sting cases in El Oued province from 2005-2020

The stings were recorded throughout the year with 72% of cases occurring from June to September. The peaks were observed in September followed by July and August, while the lows were detected in January and December with undoubted seasonality (Figure 2). Summer gathered the highest frequency (43.2%) followed by autumn (31.5%) and spring (22.3%). Most sting fatalities occurred in summer (70.2%) followed by spring (14.9%).

###  Spatial distribution of scorpion sting incidence 

**Figure 3:**
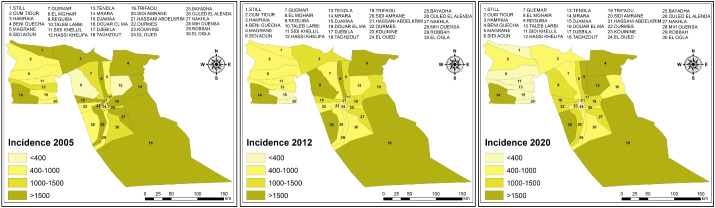
Geographical Distribution of the Incidence of Scorpion Stings for the Years 2005, 2012, and 2020

The incidence rate was calculated as the ratio of sting cases to each municipality’s population and estimated per 100 000 inhabitants. Figure 3 shows the spatial patterns of scorpion sting incidence rate for three years (2005, 2012, and 2020). Population size and the scorpion sting incidence rate were not correlated for these three years. All municipalities were affected by scorpionism at different levels. The comparison of incidence by municipality revealed a significant reduction of 70% since 2005 with *P* < 0.001. In 2005, 90% of the municipalities had an incidence greater than or equal to 400 cases per 100,000 inhabitants, of which 12 had an incidence greater than 1,500 cases per 100 000 inhabitants. The highest incidence was recorded in Hamraia with 9893 cases per 100 000 inhabitants, while the lowest reported incidence was observed in El Oued with 159 cases per 100 000 inhabitants. In 2012, 67% of the municipalities recorded a reduction in the incidence of up to 89% compared to 2005. In contrast, Reguiba municipality recorded an increase of 377%, followed by the Mih Ouensa municipality with 201% compared to 2005. For 2020, only 20% of the municipalities had recorded an incidence higher than 1,500 cases per 100 000 inhabitants. Douar El Ma and Mrara municipalities recorded a drop of 69% and 51% in the incidence compared to 2005, respectively. On the other hand, an increase of 197% was recorded for the municipality of Mih Ouensa compared to 2005. Beni Guecha, Douar El Ma, and Mrara municipalities recorded an incidence of more than 1,500 cases for the three years.

###  Statistical analysis and forecasting

 The monthly dataset included 180 observations from January 2005 to December 2019, of which the first 144 (80%) observations from 2005 to 2016 were used for model identification, and the remaining 36 (20%) observations from January 2017 to December 2019 were utilized for model validation. Data from January 2020 to December 2020 were employed for predictions.

 Examining the relationship between monthly scorpion sting cases (S) and monthly climate variables demonstrated that S was strongly positively correlated with T, Tmax, and Tmin (r was greater than 0.87, thus confirming the increase in scorpion activity with the increase in temperature), and highly negatively correlated with RH and SLP (r = -0.68 and r = -0.64, respectively). As regards the precipitations, the correlation was not significant (r = 0.014); however, it was observed that the number of scorpion stings increases after each period of heavy rainfall.

###  Multiple linear regression 

First, the ordinary least square method was performed to model the relationship between the dependent variable S (monthly scorpion sting notifications) and the explanatory variable climate factors. Variables retained for the final model after checking for the absence of multicollinearity were Tmin, Pr, and an associated β coefficient. However, the model violates the constant variance assumption; there was no autocorrelation in the residuals generated by the regression model. The weighted regression model was investigated to stabilize the variance. The model outputs are provided in Table S1 (Supplementary file 1). The outputs of the selected model indicate that the selected climate variables could explain 84.5% of the variation in the dependent variable. The resulting F-value of 488.782 is significant at *P* < 0.001, indicating that we have less than a 0.1% chance of being wrong that the model is better at explaining the number of scorpion stings.

 The estimated expression of the model is given by the following equation:

Y_predit_ = - 166.35 + 41.04 Tmin + 3.09 Pr

Tmin has a significant effect on scorpion stings (t-value 31.135, *P* < 0.0001); every unit increase in Tmin will result in an increase of 41 cases in scorpion stings. The model shows low variability between forecasted and actual values which are strongly correlated (r = 0.919).

###  Seasonal autoregressive integrated moving average model

Autoregressive integrated moving average (ARIMA) model can best be applied to data that are stable or exhibit a consistent pattern over time and with minimum outliers. The training data set exhibits a non-stationary variance. Moreover, the Box plot with a monthly average of recorded scorpion sting cases (Figure 2) displays a clear seasonality in data. To achieve stationary conditions, the logarithm was applied to stabilize the variance. A seasonal differencing was required (𝐷 = 1) given that peaks are observed at lags of 12 months (Figure S1, Supplementary file 1). The transformed time series (Figure S1, Supplementary file 1), named *sscorptr*, is stationary as confirmed by the augmented Dicky-Fuller test (t-statistic = -3.745 and *P* = 0.023). The identification of candidate values for p, q, P, and Q orders was achieved using the structure of the autocorrelation and partial autocorrelation functions (Figure S1, Supplementary file 1). The SARIMA (2.0.2)(1.1.1)_12_ model, fulfilling all the qualifications of the best model, was retained after several trials. The model equation is as follows:


$$Z_{t}=-1.255Z_{t-1}-0.556Z_{t-2}+0.446Z_{t-12}+\varepsilon_{t}+1.708\varepsilon_{t-1}+0.943\varepsilon_{t-2}-0.909\varepsilon_{t-12}$$

where *Zt = sscorptr =*∇_12_ log*(S). *All parameter values of the model are statistically significant at 5% (Table S2, Supplementary file 1). The residuals are normally distributed as confirmed by the Shapiro-Wilk normality test (W = 0.982 and *P* = 0.05371) and white noise as confirmed by the Box-Pierce test (χ^2^ = 18.013; df = 24; *P* = 0.8024). The model shows low variability between forecasted and actual values with a strong correlation (r = 0.971). Thus, the model can be used to predict the number of scorpion envenomation cases for the period from January 2020 to December of the same year. The predicted values represented significant and considerable agreement with the actual data and a strong correlation (r = 0.986).

Climate variables were included as covariates in the SARIMA (2,0,2)(1,1,1)_12_ model to assess whether the model fit and predictive power could be improved. The SARIMAX(2.0.2)(1.1.1)_12_ with Tmin at lag 0 was retained as the optimal model. The measures of accuracy of the best selected SARIMAX model (RMSE = 0.357, MAE = 0.287, and MAPE = 5.643) were lower than those of the SARIMA model (RMSE = 0.219, MAE = 0.163, and MAPE = 3.150). In addition, the SARIMA model achieved better prediction accuracy for 2020 than the SARIMAX model (SARIMAX outputs are given in Table S3, Supplementary file 1).

### NNAR model and hybrid SARIMA-NNAR model

The NNAR model was developed to account for nonlinearity. NNAR (1,1,2) with y_t-1_, y_t-12_ delays and two neurons in the hidden layer was selected as the best fitting model. Therefore, 132 values were compared in the training set.

The hybrid SARIMA-NNAR model was considered to account for both linearity and nonlinearity and seasonality in the data. The best-fit hybrid model was combined with a SARIMA (2,0,2)(1,1,1)_12_ and NNAR(1,1,2) with y_t-1_, y_t-12_ delays and two neurons in the hidden layer.

###  Comparison of models

The fitted, forecasted, and predicted values were strongly correlated with actual values (r > 0.879) for all models (Table 1).

**Table 1:** Comparison of the fitting, forecasting, and prediction accuracy of the models

**Model**	**Training Set**				**Test Set**				**Prediction 2020**			
	**RMSE**	**MAE**	**MAPE**	**r**	**RMSE**	**MAE**	**MAPE**	**r**	**RMSE**	**MAE**	**MAPE**	**r**
Multiple regression	0.911	0.506	11.71	0.879	0.986	0.585	13.39	0.931	0.518	0.355	7.488	0.894
SARIMA	0.219	0.163	3.150	0.984	0.286	0.238	4.596	0.971	0.221	0.181	3.532	0.986
NNAR	0.438	0.368	6.633	0.983	0.438	0.368	6.633	0.932	*0.204*	*0.180*	*3.368*	0.986
SARIMA-NNAR	0.211	0.165	3.201	0.984	0.289	0.235	4.557	0.971	0.242	0.192	4.003	0.984

*Note*. RMSE: Root mean square error; MAE: Mean absolute error; MAPE: Mean absolute percentage error; r: Pearson product-moment correlation coefficient.

 The training set was used taking into account lost values for each built model to compare the fitting accuracy of the three models. Therefore, for the SARIMA (NNAR and SARIMA-NNAR, respectively) model, 168 values were utilized to compare the fitting accuracy as the first 12 values in the training set were lost after the performance of a seasonal difference to the original time series to achieve the stationary.

**Figure 4:**
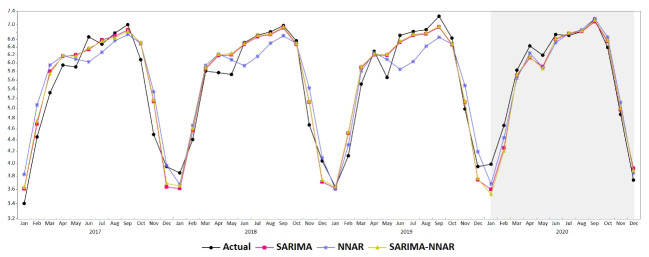
Actual, SARIMA, NNAR, and hybrid SARIMA-NNAR monthly forecasts and predictions (shaded) of monthly scorpion stings

For the NNAR model, 132 values were compared for fitting accuracy since 12 time-lagged variables were created as input features, and 168 values were compared for the fitting accuracy of the SARIMA-NNAR model. The comparison of the forecast performance for the three models was achieved using the test data set. All error measurements are presented in Table 1. It is shown that SARIMA and SARIMA-NNAR training and test set accuracy are slightly different and better than NNAR; however, the NNAR model achieved better prediction accuracy for 2020 than the SARIMA model and the hybrid model (Figure 4).

## Discussion

Although Algeria has a scorpion envenomation monitoring system which has undergone several improvements since its creation in 1986, scorpion envenomation remains an actual health problem in high plateaus and the Sahara Desert. Nearly 50,000 scorpion stings and 50 related deaths are reported every year. The country has climatic and environmental conditions conducive to the proliferation of scorpions and is home to 49 scorpion species, 20% of which are potentially dangerous to humans.^1^

 The geography, environment, climate, and man contribute to the endemicity of scorpion envenomation in El Oued province. Although the surveillance program has played a role in the decrease of both incidence and lethality, the incidence remains high. In 2020, 689 cases per 100 000 people and 2920 cases per 100 000 people were reported at the province level and as the highest incidence at the municipality level, respectively.

 The current study aimed to step up the prevention and control of the scorpion sting issue in El Oued province and help public officials make more informed decisions through the evaluation of the epidemiological profile, the determination of the spatial distribution and the seasonal pattern, and the development of models that could accurately predict future scorpion sting cases.

Over the past 16 years, the annual number of reported cases of scorpion stings in El Oued province has ranged from 5000 to 7000, and over the last decade, the incidence has hovered around 879 cases per 100 000 inhabitants, which is well in excess of that estimated country’s incidence.^5^ Some provinces in Algeria and some affected regions in Iran and Turkey recorded incidences falling within this range and even higher.^8,9^ Males represented slightly more than two-thirds of reported cases; a similar pattern is observed in the affected provinces of the country.^10-12^ Similar conclusions have been reached in studies performed in Iran and Morocco.^13,14^ However, this is not a strict rule; Grande do Norte state in Brazil showed a female predominance.^15^ The age group most affected by scorpion stings was 15-49 years with 61.9% of cases. This was also the case in Biskra province; however, varied distributions of stings by age group have been documented in other regions in the world, confirming the geographical variation in epidemiological indicators as reported by Chippaux and Goyffon.^4,16^ The number of deaths significantly decreased from 12 cases in 2006 to 2 cases in 2020. The decrease in fatal cases is due to the performance of health services that have worked to ensure adequate medical care for victims at the level of hospitals and health proximity in addition to the availability of anti-scorpionic serotherapy available free of charge in the country’s health structures, as well as bringing health services closer to citizens in remote areas. The majority of deaths were attributed to the use of traditional methods and delays in getting to public health facilities.

Children under 15 years of age represented 37% of the population of EL Oued province and accounted for 23% of sting cases and 54% of fatalities, confirming that young age is a major determinant of envenoming severity as mentioned in several studies.^4,17,18^ The high morbidity and fatality rates in children have generally been associated with the vulnerability of the immune system and the ratio of venom dose to the body weight of the patient.^19^ Children under 7 years of age are at high risk because their immune system is still developing; the risk of dying from scorpion venom is extremely greater. Many researchers have reported multiple organ failures in children envenomed by scorpions.^20,21^ This may be due to the fact that for the same amount of venom injected, children will have higher serum toxin levels than adult victims.^17^ Poor prognosis is clearly associated with delays in determining severity and in specific treatment with scorpion antivenom regardless of age group.^22^ Death often occurs as a result of factors related to the stung child and those around him, who sometimes do not react quickly, making unnecessary gestures to treat the affected area.

Stings were unevenly distributed over both space and time; all municipalities were affected at different levels. In 2020, no municipality was spared and 14 of the province’s 30 municipalities had an incidence greater than 1000 cases per 100 000 inhabitants. Uneven distribution of scorpion stings at the municipality level and the high rate of scorpion stings in some municipalities were also observed in affected provinces such as Biskra, El Bayadh, and M’Sila.^10,16,23^

Many studies reported climate as the determinant of scorpion distribution and scorpion envenomation.^1,24,25^ Temperature and relative humidity were cited in many undertaken studies as being highly correlated with scorpion stings.^5,24-30^ High sting frequencies have been reported in warm seasons and stings exhibit a seasonal pattern.^23,24,27^ A rate of 62% of scorpion stings occurred in El Oued province from June to September with a mortality rate, corresponding to the same period of 79%, which is in coherence with undertaken studies in affected regions in the world.^28,29^

Most of the mathematical approaches aimed at analyzing the collected scorpion sting data have relied on descriptive statistics.^12^ First, the association between scorpion sting cases and climate variables, using monthly data, in many affected regions, was performed using multiple linear regressions.^23,25,30^ Other studies have been conducted using simple statistical analyses and correlations.^12,30^ In recent years, other statistical approaches such as times series analysis and count data are taking over.^10,16^ A first study on the daily scorpion sting count was achieved in 2020 by Boubekeur et al.^11^ In this study, NNAR and hybrid SARIMA-NNAR models were first used in the prediction of monthly scorpion sting cases. These models can be more suitable for capturing nonlinearity in the data and can generate better predictions; numerous studies on health-related data have pinpointed the usefulness of these models to obtain appropriate predictions with high accuracy.^6,31,32^

In the present study, the multiple regression model, SARIMA, SARIMAX, NNAR, and hybrid SARIMA-NNAR models were built using monthly scorpion sting data from El Oued province. All models fit scorpion sting data in both the training and forecast process reasonably well. The multiple regression model demonstrated that any increase in minimum temperature leads to an increase in the number of scorpion stings; moreover, the predictions showed significant and strong concordance with the actual data. Being cold-blooded arthropods, scorpions are affected by the temperature of the environment.^1,24^ This is in line with the results obtained using multiple regression by Chowell et al in the state of Colima, by Molaee et al in the Dezful Area of South Western Iran, and by Ebrahim et al in Hadj Abad South Iran.^24,25,27^ The SARIMA model was developed with the response variable depending only on its previous values. The SARIMA(2.0.2)(1.1.1)_12_ model was found appropriate for forecasting data from El Oued province. The SARIMA model was also found to best fit scorpion sting data in Biskra province, Algeria, as well as Southern Iran.^16,27^ As some climate variables were significantly correlated with scorpion stings, the SARIMAX model was also built; however, all error measurements of the best selected model were far lower than those of SARIMA. To account for nonlinearity, NNAR was considered, and the NNAR(1,1,2) with y_t-1_, y_t-12_ delays and two neurons in the hidden layer was selected as the best fitting model. Lastly, the hybrid SARIMA-NNAR models were investigated to account for both linearity and nonlinearity, and seasonality in the data. The best-fit hybrid model was combined with a SARIMA (2,0,2) × (1,1,1)_12_ and NNAR neural network with y_t-1 _, y_t-12_ delays and two neurons in the hidden layer. It is shown that the performance measure values in both training and test sets of SARIMA and SARIMA-NNAR models are slightly different and better than those of the NNAR model. The slight differences between the metric measures for SARIMA and SARIMA-NNAR models for both training and test sets were also noted in a study by Akermi et al.^6^ The monthly predicted values for year 2020 demonstrated a considerable agreement with the actual data with a very strong correlation: r = 0.90 for multiple regressions, r = 0.986 for SARIMA model, r = 0.986 for NNAR model, and r = 0.984 for SARIMA-NNAR hybrid model. The NNAR model achieved better prediction accuracy for 2020 than both SARIMA and hybrid models (Table 1 and Figure 4). This study supports the conclusion reached by Davidescu et al on the performance of NNAR for the out-of-sample dataset,^33^ which contradicts the conclusions drawn in studies by Akremi et al and Maleki et al, where the model NNAR gave worse predictions than either SARIMA or SARIMA-NNAR model.^6,32^

 The present study has some limitations. Human actions on the environment, extent of urban construction, circumstances of accidents, and first-aid practices were not considered due to limited data availability.

 Developing and implementing predictive models into the surveillance system could be of great help to health policymakers in the development of informed, effective, and targeted policies and to initiate rapid response measures to unusual situations. Additional efforts should focus on reducing seasonal incidence by organizing training sessions on resuscitation for the benefit of general practitioners practicing in remote communities for proper management of the victims of scorpion envenomation. In addition to raising the awareness of different social categories to the rules of environmental hygiene, public outreach campaigns need to be stepped up through social or community networks.

**Highlights**

- Overall, 96909 scorpion sting cases were recorded in El Oued province from 2005-2020.
- The most affected age group was 15-49 years, and males were more likely to be stung. More than half of reported deaths belonged to children 15 and younger.
- The incidence rate ranged from 590 (2017) to 1315 (2007) per 100 000 inhabitants.
- Scorpion activity was conditioned by climate factors; temperature had the highest effect.
- The neural network autoregressive model was preferred for short-term monthly scorpion sting predictions with high accuracy.

## Conclusion

 Scorpion stings occurred throughout the year with peaks in September followed by July and August and troughs in December and January. Sting cases were not evenly distributed across demographic groups; the most affected age group was 15-49 years, and males were more likely to be stung. More than half of reported deaths belonged to children 15 and younger. Stings were unevenly distributed across municipalities. The NNAR model was preferred for short-term monthly scorpion sting predictions and produced better predictive accuracy compared to SARIMA and SARIMA-NNAR models. An in-depth understanding of the epidemiologic triad of scorpionism and the development of predictive models ought to establish enlightened, informed, better-targeted, and more effective policies.

## Acknowledgements

 We would like to thank the DHP of El Oued province for providing the necessary data.

## Authors’ Contribution

**Conceptualization:** Mohamed L’Hadj, Schehrazad Selmane, Safia Zenia.

**Data curation:** Schehrazad Selmane, Safia Zenia.

**Formal analysis:** Schehrazad Selmane, Safia Zenia.

**Investigation:** Mohamed L’Hadj, Schehrazad Selmane, Safia Zenia.

**Methodology:** Mohamed L’Hadj, Schehrazad Selmane, Safia Zenia.

**Project administration:** Schehrazad Selmane.

**Resources:** Mohamed L’Hadj, Schehrazad Selmane, Safia Zenia.

**Software:** Schehrazad Selmane, Safia Zenia.

**Supervision**: Schehrazad Selmane.

**Validation:** Mohamed L’Hadj, Schehrazad Selmane, Safia Zenia.

**Visualization:** Mohamed L’Hadj, Schehrazad Selmane, Safia Zenia.

**Writing–original draft:** Mohamed L’Hadj, Schehrazad Selmane, Safia Zenia.

**Writing–review & editing:** Mohamed L’Hadj, Schehrazad Selmane, Safia Zenia.

## Competing Interests

 The authors declare that they have no conflict of interests.

## Funding

 None.

## Supplementary Files

Supplementary file 1. Description of utilized statistical approaches and model outputs
