# Supplementary material for: A Hybrid Approach Based on Seasonal Autoregressive Integrated Moving Average and Neural Network Autoregressive Models to Predict Scorpion Sting Incidence in El Oued Province, Algeria, From 2005 to 2020
Source: J Res Health Sci. 2023 Sep 29;23(3):e00586. doi: 10.34172/jrhs.2023.121 (PMC10660509; doi:10.34172/jrhs.2023.121)
Supplement: Supplementary file 1 — Description of utilized statistical approaches and model outputs [file jrhs-23-e00586-s001.pdf]

doi:10.34172/jrhs.2023.121
JRHS 2023; 23(3):e00586

Supplementary file 1

Seasonal Autoregressive Moving Average Model

The Box-Jenkins approach, also known as Autoregressive Integrated Moving Asverage (ARIMA) models, for the analysis of time series data is one of the most widely used predictive techniques in epidemiological surveillance.

Given a stationary time series of data $X_t(t = 1, \ldots, n)$, the SARIMA model, denoted by $(p, d, q)(P, D, Q)_S$, can be expressed by the following difference equation:

$$\phi_p(B)\Phi_P(B^S)\nabla^d\nabla_S^D X_t = \theta_q(B)\Theta_Q(B^S)\varepsilon_t$$

where the backward shift operator B is defined as $B^k X_t = X_{t-k}$. and S represents the seasonality period. In addition, d, D, and p denote the number of nonseasonal differences, the degree of seasonal integration, and the number of AR terms, respectively. Further, P, q, and Q are the degree of seasonal AR terms, the number of MA terms, and the degree of the seasonal MA model, respectively. Furthermore, ∇=1−B, ∇_s= $1 - B^s$, and ε_t denote the differencing operator, the seasonal differencing operator, and the white noise process, respectively. The polynomials $\phi_p(B), \Phi_P(B), \theta_q(B), \Theta_Q(B)$ are the AR, the MA, the seasonal AR, and the seasonal MA polynomials, respectively.

SARIMA modelling is best performed while following a protocol. The first step is to check the stationary condition. The augmented Dickey-Fuller unit-root test was used for this purpose. To stabilize the variance of a time series that exhibits non-stationary variance, transformations such logarithm, square root, or reciprocal can be applied to each observation $X_t(t = 1, \ldots, n)$. To stabilize the mean, an appropriate order of differences can render a non-stationary series a stationary one. The orders p and q are lags for cutting off the autocorrelation function and partial autocorrelation function, respectively. Once orders are determined, the parameters may be estimated by a nonlinear optimization technique or the least squares procedure. The residuals are analyze to test the model for goodness-of-fit. The residuals should be uncorrelated with a mean of zero and follow a Gaussian distribution; moreover, the autocorrelations of the residuals should not be significantly different from zero. The correlation structure provides various choices for p and q values, thus generating several models. The best-fit model selection is based on criteria such as the smallest Akaike information criterion (AIC), smallest Schwarz Bayesian

information criterion (BIC), smallest Root Mean Squared Error (RMSE), smallest Mean Absolute Error (MAE), smallest Mean Absolute Percentage Error (MAPE), and the highest adjusted R^2 in addition to stationary and invertibility conditions and the white noise condition for residuals.

Neural Network Model

Given the observed nonlinear trend in the data, ANN is one among the appropriate models that can be used to approximate various nonlinearities in the data. The single hidden layer feed forward network is the most widely applied model form for time series modeling and forecasting. This model is characterized by a network of three layers, namely, input layer (Input variables), hidden layer (Layers of nodes between the input and output layers), and the output layer (output variables) of simple processing units which are connected by acyclic links.

The relationship between the output y_t, and $y_{t-1}, y_{t-2}, \dots, y_{t-p}$ is formalized as follows:

$$y_t = \alpha_0 + \sum_{j=1}^{q} \alpha_j g\left(\beta_{0j} + \sum_{i=1}^{p} \beta_{ij} y_{t-i}\right) + \epsilon_t$$

where p and q are the number of input nodes and the number of hidden nodes, respectively. Moreover, $\alpha_j (j = 0,1, \dots, q)$ and $\beta_{ij} (i = 0,1, \dots, p; j = 1, \dots, q)$ represent the parameters of the model, and g denotes the hidden layer transfer function. The logistic function defined by

$$g(x) = \frac{1}{1 + e^{-x}}$$

was utilized as the hidden layer transfer function. It is noteworthy that the neural network and non-linear AR model have similar representation.

Hybrid SARIMA-NNAR Forecasting

Almost all real-world time series contains both linear and non-linear correlation structures among the observations. Neither ARIMA nor ANN is universally suitable for all types of time series. Indeed, the approximation of nonlinear time series by ARIMA models or linear time series by ANN models may not be appropriate. The $NNAR(p, P, k)_m$ model was employed in this study. It is one type of the ANN model, in which the lagged values of data can be used as inputs to the neural network. An $NNAR(p, P, k)_m$ model has inputs $(y_{t-1}, y_{t-2}, \dots, y_{t-p}, y_{t-m}, y_{t-2m}, \dots, y_{t-Pm})$ and k neurons in the hidden layer. A

$NNAR(p,P,0)_m$ model is equivalent to an $ARIMA(p,0,0)(P,,0,0)_m$ model but without restrictions on parameters that ensure stationarity. The hybrid method is proposed for combining the linear and nonlinear models. To perform this method, the original time series at time t needs to be composed of an auto-correlated linear (L_t) and a nonlinear (N_t) components.

First, the SARIMA model is utilized to capture the linear component in the data. Thereafter, NNAR is used to capture the nonlinear component in the residuals part. The residuals are expressed as $e_t = y_t - \hat{L}_t$, where $\hat{L}_t$ is the forecasting value at time t of y_t, estimated by the SARIMA model, and are represented as follows:

$$e_t = f\left(e_{t-1}, e_t e_{t-2}, \ldots, e_{t-p}\right) + \epsilon_t = \widehat{N}_t + \epsilon_t$$

where p and ϵ_t represent the optimal number of lags and the white noise, respectively. Additionally, $\widehat{N}_t$ denotes the forecast value at time t by the NNAR model, and f is a nonlinear function determined by the multilayer perceptron.

The linear and nonlinear forecasting values obtained by SARIMA and NNAR models are then combined to get the forecast:

$$\hat{z}_t = \hat{L}_t + \widehat{N}_t.$$

Measures of Accuracy

Frequently used metrics to measure performance and estimate the accuracy of the forecasts and to compare different models are:

Root Means Square Rrror: $RMSE = \sqrt{\frac{1}{n}\sum_{t=1}^{n} \left(X_t - \hat{X}_t\right)^2}$

Mean Absolute Error: $MAE = \frac{1}{n}\sum_{t=1}^{n} \left|X_t - \hat{X}_t\right|$

Mean Absolute Percentage Error: $MAPE = \frac{1}{n}\sum_{t=1}^{n} \frac{|X_t - \hat{X}_t|}{X_t}$

where n, $\hat{X}_t$, and X_t are the size of the test set, the forecasted observation, and the actual observation at time t, respectively. Model with the lowest value of the error measurements indicates the better performance model.

Akaike Information Criterion and Schwarz Bayesian Information Criterion

Akaike information criterion (AIC)

$$AIC_{p,q} = n * \ln(MSE) + 2k$$

The Schwarz Bayesian information criterion (BIC)

BIC = $n * \ln(MSE) + k * ln(n)$

where n is the number of observations (sample size) of the time series used to build the model. In addition, ln and k represent the natural logarithm and the number of model parameters k=p+q+P+Q+1, respectively.

Minimum *AIC* and *BIC* values are utilized as model selection criteria. Models were built using TSA package, forecast package, neuralnet package, and the nnetar function in the forecast package for R to fit a neural network model to a time series with lagged values of the time series as inputs and forecast hybrid package under R software version 3.4.4 (Network Theory Ltd., Bristol, UK).

Multiple Regression Analysis Results

Table S1. Weighted Least Square Regression Model

Factor	β	S.E	T-value	*P*-value		VIF
Constant	-166.345	16.831	-9.884	0.001		
Tmin	41.04	1.318	31.135	0.001		1.001
Pr	3.092	1.841	1.68	0.095		1.001
r	R^2	Adjusted R^2	SE	F-value	*P*-value	Durbin-Watson
0.919	0.847	0.845	1.228	488.782	0.001	1.429

Note. VIF: Variance inflation factor; SE: Standard error; SARIMA: Seasonal autoregressive integrated moving average. SARIMA outputs.

Table S2. Parameter Estimates of Seasonal Autoregressive Integrated Moving Average Model

	ar1	ar2	ma1	ma2	sar1	sma1
Coefficient	-1.255	-0.556	1.708	0.943	0.446	-0.909
t-stat	-13.708	-5.446	35.355	14.895	2.530	-3.708
P-value	0.001	0.001	0.001	0.001	0.011	0.001

Note. Akaike information criterion (AIC) = 12.89; Schwarz Bayesian information criterion (BIC) = 33.07; Log likelihood = 0.55.

SARIMAX Outputs

Table S3. Parameter Estimates of Seasonal Autoregressive Integrated Moving Average With Covariate

	ar1	ar2	ma1	ma2	sar1	sma1	Tmin
Coefficient	0.630	0.359	-0.136	-0.684	0.989	-0.620	0.071
s.e.	0.1782	0.1743	0.1363	0.0956	0.0053	0.0793	0.0104
t-stat	2.031	14.655	1.571	-14.340	30.647	-5.532	6.7834
P-value	0.001	0.040	0.319	0.001	0.001	0.001	0.001

Note. Akaike information criterion (AIC) = 34.255; Schwarz Bayesian information criterion (BIC== 55.044, Log likelihood=-10.127.

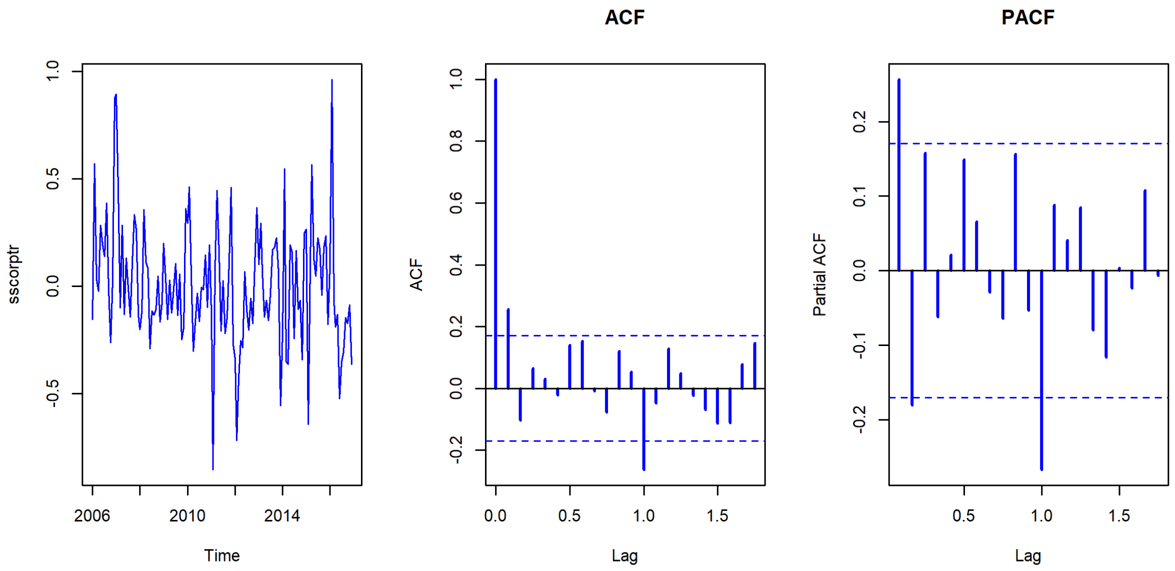

Figure S1. Transformed Monthly Time Series From 2005-2017 and Corresponding Autocorrelation Function and Partial Autocorrelation Function Plots
